# Performance trade-offs between dense prediction and sparse query mechanisms for brain tumor MRI detection: a comparative study of YOLOv8 and RT-DETR

**DOI:** 10.3389/fmed.2026.1905061

**Published:** 2026-08-31

**Authors:** Lianxin Xie, Jinghui Chen, Zhipeng Sun, Yuhan Huang, Shan Ye, Chengbin Ye

**Affiliations:** 1 The First Clinical Medical College, The Affiliated People's Hospital of Fujian University of Traditional Chinese Medicine, Fuzhou, Fujian, China; 2 Fujian Medical University, Fuzhou, China; 3 Department of Medical Imaging, The Affiliated People's Hospital of Fujian University of Traditional Chinese Medicine, Fuzhou, Fujian, China

**Keywords:** artificial intelligence, brain tumor, hyper-parameter optimization, magnetic resonance imaging (MRI), object detection, performance trade-off, RT-DETR, YOLOv8s

## Abstract

**Objective:**

This study investigates optimal training strategies for YOLOv8s in brain tumor MRI detection, systematically evaluates the effects of key hyperparameters on model performance, and compares the adaptability and trade-offs between convolution-based dense prediction and Transformer-based sparse query mechanisms in medical image detection.

**Methods:**

Experiments were conducted on publicly available Kaggle MRI datasets of meningioma and glioma. YOLOv8s was adopted as the baseline model, and a systematic hyperparameter search was performed over learning rates (0.01, 0.001, and 0.0001) and bounding box regression loss weights (5.0, 7.5, and 10.0), forming nine configurations evaluated via five-fold cross-validation. Based on the optimal setting (learning rate = 0.01, loss weight = 7.5), the original YOLOv8s detection head was replaced with an RT-DETR Transformer-based decoder head. Performance and generalization were assessed on both internal and external test sets, and Score-CAM was employed for model interpretability analysis.

**Results:**

The hyperparameter analysis demonstrated that the learning rate was the primary factor affecting detection performance, whereas the influence of the bounding box regression loss weight was relatively limited within a reasonable range. Using the optimal hyperparameter combination (learning rate = 0.01 and box loss weight = 7.5), the native YOLOv8s detection head achieved the best overall performance, with a precision of 91.7%, a recall of 89.2%, and mAP@50 of 92.6%, outperforming the RT-DETR detection head (mAP@50 = 91.1%). On the external test set, YOLOv8s also demonstrated superior cross-domain generalization (mAP@50 = 79.0%), exceeding RT-DETR by 10.1%. Further NMS IoU sensitivity analysis showed that the RT-DETR detection head maintained more stable performance across different NMS IoU thresholds, whereas YOLOv8s was more sensitive to variations in NMS post-processing. Score-CAM visualization further revealed distinct attention patterns, with YOLOv8s exhibiting broader lesion responses and RT-DETR focusing more selectively on lesion regions.

**Conclusion:**

Appropriate hyperparameter optimization can substantially improve the performance of brain tumor MRI detection models. The dense prediction mechanism of YOLOv8s provides superior lesion coverage, resulting in better detection performance and cross-domain generalization, whereas the sparse query mechanism of RT-DETR exhibits greater robustness to NMS parameter variations during inference. These findings highlight the complementary characteristics of the two detection paradigms and provide practical guidance for detection head selection and inference strategy optimization in medical object detection.

## 1 Introduction

Brain tumors are among the most serious malignancies threatening human health, and their high mortality and disability rates continue to drive extensive research efforts worldwide. Epidemiological studies have shown that although brain tumors account for only approximately 1% of all malignant neoplasms, they contribute to nearly 3% of cancer-related deaths, imposing a substantial burden on patients' quality of life (1). Among the various types of brain tumors, gliomas, and meningiomas are the two most prevalent categories. Gliomas are highly aggressive and typically exhibit infiltrative growth patterns with irregular boundaries, resulting in poor clinical outcomes. In contrast, meningiomas are predominantly benign; however, their anatomical locations often complicate surgical resection, making accurate preoperative localization critically important. Therefore, the early and precise detection of brain tumors is essential for treatment planning and prognosis improvement.

Magnetic resonance imaging (MRI) has become the primary imaging modality for brain tumor detection and diagnosis owing to its superior soft-tissue contrast compared with computed tomography (CT) (2, 3). MRI enables accurate differentiation between tumor tissue and normal brain structures across multiple imaging sequences, providing crucial information for tumor classification, grading, treatment planning, and prognostic assessment. Nevertheless, in routine clinical practice, MRI examinations generate large volumes of imaging data, and the radiological manifestations of brain tumors are highly heterogeneous. Tumors with different pathological subtypes and grades often exhibit substantial variations in morphology, signal intensity, and boundary definition, placing considerable demands on the expertise of radiologists. Currently, clinical diagnosis largely relies on manual slice-by-slice image interpretation and visual identification of tumor lesions. This process is labor-intensive and time-consuming, resulting in limited diagnostic efficiency and susceptibility to inter-observer variability, subjective judgment, and fatigue-related errors. Furthermore, the detection accuracy of small early-stage tumors or lesions with indistinct boundaries remains particularly challenging. Consequently, the development of an efficient and accurate automated brain tumor MRI detection approach is of substantial clinical significance for assisting radiologists, improving diagnostic efficiency, reducing missed diagnoses, and facilitating the implementation of precision medicine.

With the rapid advancement of artificial intelligence, deep learning–driven medical image analysis has profoundly transformed traditional diagnostic paradigms. Owing to their powerful capability for hierarchical feature extraction, convolutional neural networks (CNNs) have achieved remarkable success in a wide range of medical imaging tasks, including image classification and object detection. In the field of brain tumor MRI detection, numerous deep learning–based automated detection approaches have been proposed, and their performance has been continuously improved through the incorporation of various optimization strategies and architectural enhancements (4). However, two-stage detection frameworks represented by Mask R-CNN suffer from inherent limitations in inference speed and computational efficiency, making them less suitable for real-time clinical applications. These limitations become even more pronounced when deployment is required on resource-constrained medical devices (5).

In the field of object detection, the YOLO (You Only Look Once) series of frameworks has demonstrated remarkable advantages in balancing detection accuracy and inference speed since its introduction in 2016 (6). Benefiting from its efficient single-stage end-to-end detection paradigm, YOLO has become an important technical approach for real-time medical image analysis and has been increasingly adopted in various computer-aided diagnostic applications.

As a major advancement within the YOLO family, YOLOv8 incorporates several architectural innovations that further improve detection performance and model efficiency. Compared with YOLOv5, YOLOv8 adopts an anchor-free detection paradigm, eliminating the dependence on predefined anchor boxes and enhancing adaptability to objects of varying scales (7). In addition, its decoupled detection head separates classification and localization tasks, thereby reducing task interference and facilitating more effective optimization of individual objectives (8). Furthermore, the improved C2f backbone module enhances feature reuse while maintaining a lightweight architecture, leading to stronger multi-scale feature representation capabilities. Collectively, these architectural improvements have enabled YOLOv8 to achieve superior performance on general object detection benchmarks while retaining a relatively compact model size, making it a promising candidate for deployment in resource-sensitive medical imaging applications. Nevertheless, the full potential of YOLOv8 for brain tumor MRI detection remains insufficiently explored. In particular, systematic investigations into task-specific hyper-parameter optimization are still limited, despite their critical role in determining model performance and generalization capability.

The performance of deep learning models is critically governed by hyper-parameter configuration, and tuning remains a fundamental challenge in practical deep learning engineering, as optimal hyper-parameter selection directly enhances model generalization (9). Among the numerous hyperparameters involved in model training, the learning rate and loss function weighting strategy exert particularly significant influences on both the optimization process and the final detection performance. As a key parameter governing the magnitude of model updates during training, the learning rate directly affects the convergence speed and convergence quality of a neural network. An excessively high learning rate may cause instability in the optimization process, leading to oscillation or even divergence of the loss function and preventing the model from converging to an optimal solution (10). Conversely, an overly low learning rate can substantially prolong the training process and increase the risk of the model becoming trapped in suboptimal local minima, thereby compromising its generalization capability (11). Previous studies have demonstrated that selecting an appropriate learning rate for a specific task and dataset is critical for achieving optimal detection performance, and that different learning rate strategies may exhibit markedly different behaviors across application scenarios (12).

In the YOLO family of object detection frameworks, the bounding box regression loss weight represents another critical training hyperparameter, as it directly regulates the emphasis placed on localization errors during the optimization process (13). Within the multi-task learning paradigm, classification loss, objectness loss, and bounding box regression loss jointly constitute the overall loss function, and the relative weighting of these components substantially influences the model's learning priorities across different tasks. In brain tumor MRI detection, the appropriate configuration of the bounding box regression loss weight is particularly important because tumor lesions often exhibit indistinct boundaries, heterogeneous morphologies, and considerable anatomical variations across pathological subtypes. Under such circumstances, accurate localization of tumor regions relies heavily on a balanced optimization strategy. If the regression loss weight is set too low, the model may fail to sufficiently optimize localization accuracy, resulting in poor overlap between the predicted bounding boxes and the ground-truth lesion regions. Conversely, an excessively high regression loss weight may suppress the optimization of classification and objectness prediction, potentially increasing the false-positive rate and compromising overall detection reliability (14).

Despite the growing adoption of YOLOv8 in medical image analysis, systematic investigations into hyperparameter optimization for brain tumor MRI detection remain limited. Most existing studies rely on default framework settings or empirically selected hyperparameters, with relatively little effort devoted to comprehensive and task-specific optimization. Consequently, the effects of different hyper-parameter combinations on key detection metrics, including Precision, Recall, and mean Average Precision (mAP), have not been fully characterized. This knowledge gap may hinder the realization of the full performance potential of YOLOv8 in brain tumor detection applications. Furthermore, the interaction between learning rate and loss weighting is inherently complex, and the isolated adjustment of a single hyperparameter often fails to reveal the optimal configuration for a specific task. Therefore, systematic comparative experiments are required to elucidate the combined effects of these hyperparameters on model performance.

In recent years, deep learning methods have been widely applied to brain tumor MRI analysis tasks. Convolutional neural network (CNN)-based approaches have achieved promising performance in brain tumor classification through hierarchical feature extraction. For instance, transfer learning models such as ResNet50V2 and DenseNet121 can effectively identify different tumor types, including meningioma (15). However, these methods are primarily designed for image-level classification and are incapable of providing precise lesion localization. In contrast, segmentation methods based on U-Net and its variants enable more refined tumor region delineation and exhibit advantages in boundary identification (16), yet they typically rely on substantial pixel-level annotated data, incurring high training costs that hinder their applicability in rapid screening scenarios. Recently, object detection models have been progressively adopted for brain tumor MRI analysis, as they can simultaneously achieve lesion localization and category prediction, demonstrating considerable potential in computer-aided diagnosis. Moreover, improved detection frameworks incorporating attention mechanisms and lightweight architectures have been developed to enhance brain tumor detection performance (17). In addition, optimization algorithm-based machine learning models and ensemble deep learning frameworks have also been employed to improve MRI classification accuracy (18).

Nevertheless, most existing studies focus primarily on improving the performance of individual models, while systematic evaluations of the applicability of different detection mechanisms for brain tumor MRI detection remain lacking. In particular, the performance disparities, generalization capabilities, and applicable conditions between convolution-based dense prediction methods and Transformer-based sparse query methods in medical image detection have not been sufficiently investigated. To address this gap, the present study, grounded in the YOLOv8s framework, systematically analyzes the influence of key training strategies on model performance and further compares the performance of convolutional dense prediction detection heads and Transformer-based sparse query detection heads in the context of brain tumor MRI detection.

To address these limitations, this study employed YOLOv8s as the baseline framework and systematically investigated the combined effects of three learning rates (0.01, 0.001, and 0.0001) and three bounding box regression loss weights (5.0, 7.5, and 10.0). A total of nine experimental groups were constructed using a full-factorial design to comprehensively evaluate the influence of different hyper-parameter combinations on Precision, Recall, and mAP@50. The objective was to establish an evidence-based optimal hyper-parameter configuration for brain tumor MRI detection using YOLOv8s and to provide a robust experimental foundation for subsequent model architecture optimization studies.

Building upon the optimal hyper-parameter setting identified through these experiments, an ablation study was further conducted by replacing the native YOLOv8s detection head with an RT-DETR-based detection head. This design enabled a systematic comparison between the convolutional dense prediction paradigm and the Transformer-based sparse query paradigm in the context of brain tumor MRI detection. By evaluating their respective performance characteristics and task adaptability, this study aims to provide empirical evidence for selecting appropriate detection mechanisms according to different clinical requirements.

The main contributions of this study are threefold. First, we systematically analyze the effects of learning rate and bounding box regression loss weight on the detection performance of YOLOv8s for brain tumor MRI, thereby identifying the training strategies best suited for this task. Second, while keeping the feature extraction network consistent, we compare the performance discrepancies between the convolutional dense prediction detection head and the Transformer-based sparse query detection head, providing an in-depth analysis of the applicability of these two detection mechanisms in medical imaging tasks.

### 1.1 Experimental methods

#### 1.1.1 Model analysis

In the field of object detection, two-stage detectors represented by Faster R-CNN generally achieve high detection accuracy; however, their sequential processing pipeline results in slow inference speed and substantial computational overhead, limiting their applicability in real-time clinical scenarios. In contrast, the YOLO family of single-stage detectors has achieved a favorable balance between detection accuracy and computational efficiency through an end-to-end unified detection framework. Nevertheless, YOLOv5 still exhibits several inherent limitations. First, its predefined anchor-based mechanism is highly sensitive to anchor scale configuration, which restricts its adaptability to brain tumors with diverse morphologies and sizes. Second, the coupled detection head shares parameters between classification and localization tasks, making the optimization process susceptible to task interference and thereby constraining further improvements in detection performance (19).

**Figure 1:**
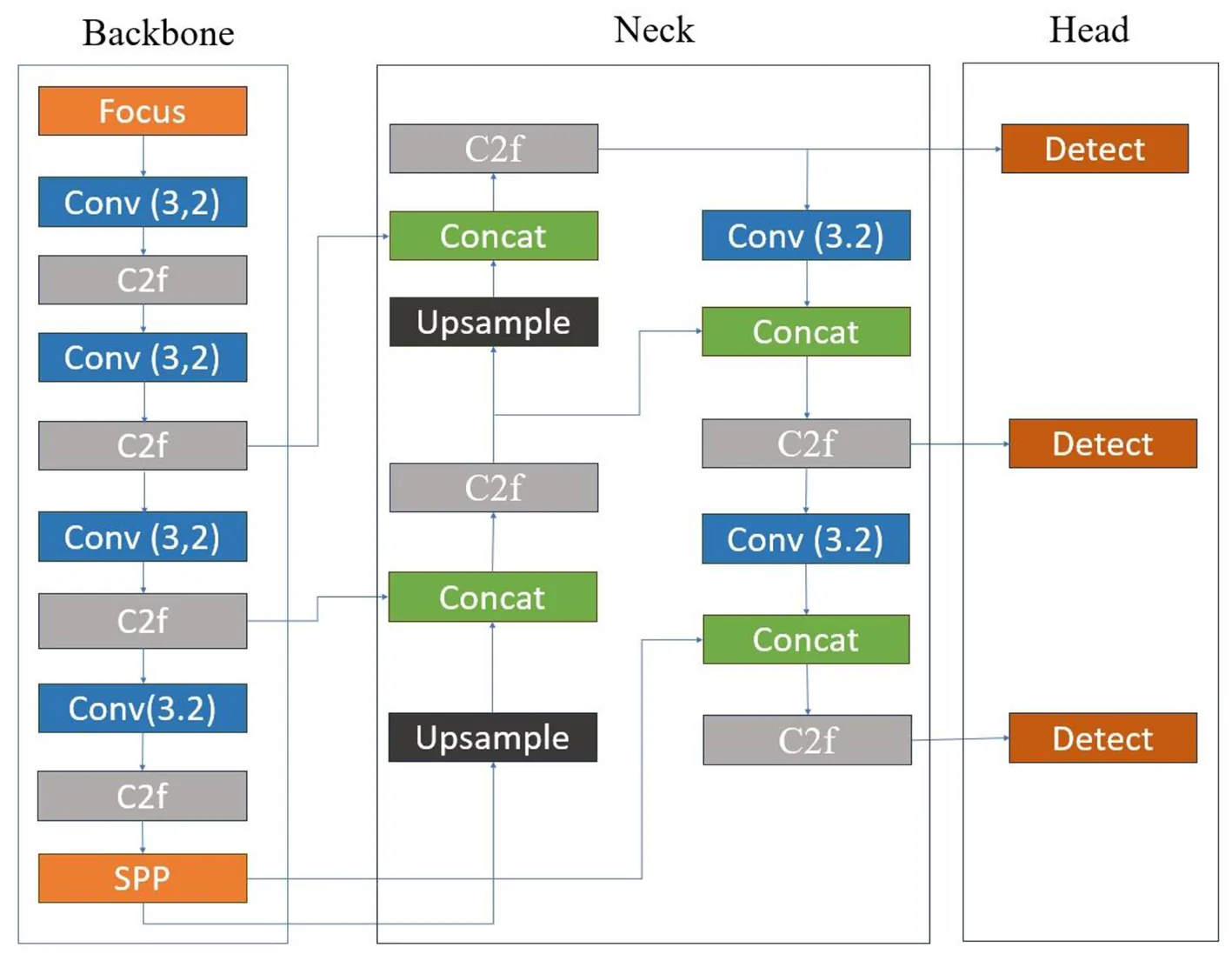
Schematic diagram of the YOLOv8s module architecture.

To address the aforementioned limitations, YOLOv8 introduces three major architectural improvements. The overall architecture of YOLOv8s used in this study is illustrated in Figure 1. First, the C3 module is replaced by the C2f module, which incorporates richer residual connection pathways to enhance feature reuse and gradient propagation efficiency. This design strengthens deep semantic feature extraction while maintaining a lightweight architecture. Second, YOLOv8 adopts a decoupled detection head that assigns classification and localization tasks to independent branches, thereby reducing task interference and enabling more effective optimization of each objective. Third, the model abandons the traditional anchor-based paradigm and instead employs an anchor-free detection strategy that directly predicts object centers and bounding box dimensions. This change substantially reduces dependence on manually predefined anchor configurations and improves adaptability to objects with diverse morphologies and scales.

Compared with the contemporaneous RT-DETR framework, which enhances global contextual modeling through a Transformer-based architecture, the relatively higher computational complexity of RT-DETR poses challenges for deployment in resource-constrained medical environments (20). By retaining the efficient inference characteristics of a pure convolutional architecture while simultaneously improving detection accuracy, YOLOv8 achieves a more favorable overall balance among inference speed, model lightweightness, and detection performance, making it a suitable foundation for automated brain tumor MRI detection.

#### 1.1.2 Hyper-parameter analysis and experimental design

The performance of a deep learning model is highly dependent on the appropriate configuration of training hyperparameters. In this study, two critical hyperparameters were investigated: the learning rate and the bounding box regression loss weight. These parameters play fundamental roles in determining the optimization dynamics of the model and ultimately influence its detection performance.

The learning rate is a key hyperparameter that governs the magnitude of parameter updates during training and directly affects both the convergence behavior and final performance of the model. In the gradient descent optimization process, model parameters are updated according to the following rule:


$$\begin{array}{l} \begin{array}{c} \theta_{t+1}=\theta_{t}-\eta \cdot \nabla_{\theta }L\left(\theta_{t}\right)\; \end{array} \end{array}$$ (1)

where θ_*t*_denotes the model parameters at iteration *t*, η represents the learning rate, and $\nabla_{\theta }L\left(\theta_{t}\right)$denotes the gradient of the loss function with respect to the model parameters. As indicated by the Equation 1, the learning rate directly determines the magnitude of parameter updates during each optimization step. When the learning rate is excessively high, the update steps may become overly large, causing oscillation or even divergence of the loss function and preventing the model from converging to a stable optimum. Conversely, an excessively low learning rate results in slow parameter updates, substantially prolonging the training process and increasing the likelihood of the model becoming trapped in suboptimal local minima, thereby impairing its generalization capability. Therefore, identifying an appropriate learning rate for a specific task and dataset is a prerequisite for fully exploiting the performance potential of a deep learning model.

The bounding box regression loss weight represents another critical hyperparameter in the YOLO family of object detection frameworks. In YOLOv8, the overall loss function is formulated as a weighted combination of multiple task-specific loss components:


$${ℒ}_{tottal}=\left(\lambda_{box}\cdot {ℒ}_{box}+\lambda_{cls}\cdot {ℒ}_{cls}+\lambda_{dfl}\cdot {ℒ}_{dfl}\right)$$ (2)

where in Equation 2, ${ℒ}_{box},{ℒ}_{cls}$, and ${ℒ}_{dfl}$denote the bounding box regression loss, classification loss, and Distribution Focal Loss (DFL), respectively, while λ_*box*_, λ_*cls*_, and λ_*dfl*_represent their corresponding weighting coefficients. In this study, particular attention was devoted to investigating the influence of λ_*box*_on detection performance. By adjusting the relative contribution of the localization objective to the overall optimization process, λ_*box*_directly affects the balance between object localization and classification learning. Specifically, YOLOv8 employs Complete Intersection over Union (CIoU) as the bounding box regression loss to optimize localization accuracy. The CIoU loss is formulated as follows:


$$\begin{array}{l} \begin{array}{c} L_{box}=1-CIoU=1-\left(IoU-\rho^{2}\left(b,b^{gt}\right)c^{2}-\alpha v\right)\; \end{array} \end{array}$$ (3)

where in Equation 3, *IoU* denotes the Intersection over Union between the predicted bounding box and the ground-truth bounding box; ρ^2^(*b, b*^*gt*^)represents the squared Euclidean distance between their center points; *c* denotes the diagonal length of the smallest enclosing box covering both the predicted and ground-truth boxes; α is a balancing coefficient; and *v* measures the consistency of the aspect ratios between the predicted and ground-truth boxes. Compared with conventional IoU-based loss functions, CIoU simultaneously incorporates three geometric factors—overlap area, center-point distance, and aspect-ratio consistency—thereby providing a more comprehensive optimization signal for bounding box regression. This property is particularly advantageous for brain tumor MRI detection, where lesions often exhibit substantial variability in shape, size, and boundary characteristics.

The weighting coefficient λ_*box*_determines the relative importance of the localization objective during model optimization. When λ_*box*_is set too low, the model may place insufficient emphasis on localization learning, resulting in poor overlap between predicted bounding boxes and the actual lesion regions. Conversely, an excessively large λ_*box*_may overemphasize localization optimization at the expense of classification and confidence-related objectives, potentially suppressing the optimization of $L_{cls}$and $L_{dfl}$, increasing the false-positive rate, and ultimately compromising overall detection reliability.

Furthermore, a potential interaction exists between the learning rate (η) and the bounding box regression loss weight (λ_*box*_). Different configurations of λ_*box*_alter the relative magnitudes of individual loss components, thereby influencing the gradient distribution of the overall loss function ($L_{total}$). As a consequence, an identical learning rate may exhibit substantially different convergence behaviors under different loss-weighting schemes. This interaction highlights that the optimization dynamics of deep learning models are governed not only by the choice of individual hyperparameters but also by their combined effects. Therefore, considering these hyperparameters in isolation is often insufficient for achieving optimal training performance (21).

Motivated by this consideration, the present study systematically investigated the combined effects of three learning rates (0.01, 0.001, and 0.0001) and three bounding box regression loss weights (5.0, 7.5, and 10.0). A total of nine experimental groups were constructed using a full-factorial design to comprehensively evaluate the interaction patterns between these two classes of hyperparameters. The ultimate objective was to identify an optimal hyper-parameter configuration for YOLOv8s in brain tumor MRI detection and to provide experimental evidence for understanding how hyper-parameter interactions influence model performance. The detailed experimental design is presented in Table 1.

**Table 1:** Experimental design of YOLOv8s hyper-parameter combinations.

Lr	Box loss weight	Research purpose
0.01	5.0	High learning rate + low regression weight
0.01	7.5	High learning rate + medium regression weight
0.01	10.0	High learning rate + high regression weight
0.001	5.0	Medium learning rate + low regression weight
0.001	7.5	Medium learning rate + medium regression weight
0.001	10.0	Medium learning rate + high regression weight
0.0001	5.0	Low learning rate + low regression weight
0.0001	7.5	Low learning rate + medium regression weight
0.0001	10.0	Low learning rate + high regression weight

#### 1.1.3 Analysis of the YOLOv8s detection head mechanism

In addition to training strategies, the architectural design of the detection framework also plays a critical role in determining model performance. After optimizing key hyperparameters, including the learning rate and bounding box regression loss weight, this study further investigates the applicability of different detection mechanisms in brain tumor MRI detection from the perspective of detection head design. Under consistent training settings and identical data splits, a comparative analysis is conducted between the original YOLOv8s detection head and the RT-DETR detection head to examine how different object detection paradigms influence brain tumor lesion localization performance.

**Figure 2:**
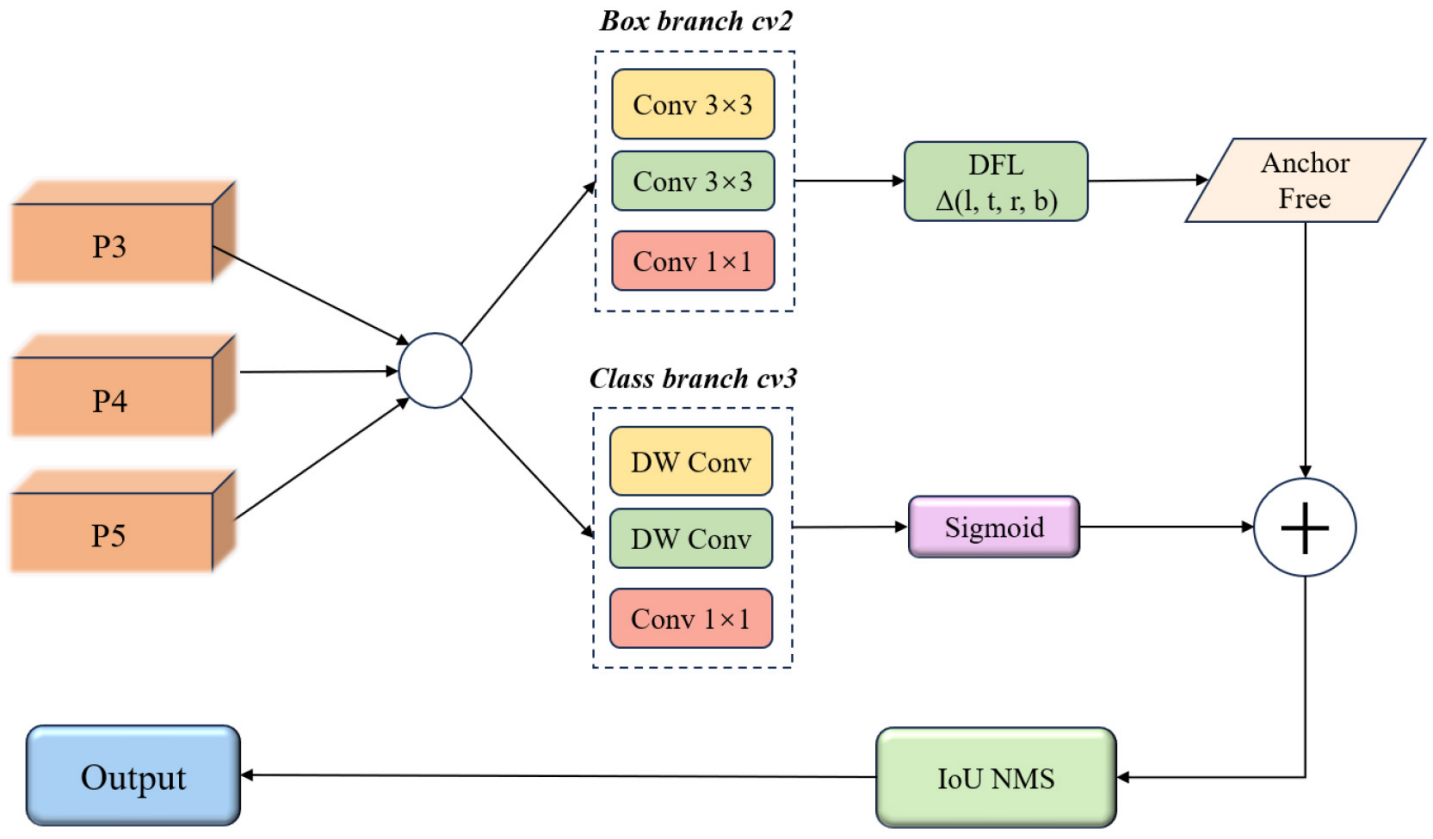
Schematic diagram of the native YOLOv8s detection head structure.

Figure 2 illustrates the structure of the native YOLOv8s detection head. YOLOv8s adopts a convolutional neural network (CNN)-based dense prediction detection paradigm, in which the detection head performs dense regression over all spatial locations of the feature maps to jointly predict object categories and bounding boxes (22). Compared with traditional anchor-based object detection frameworks, YOLOv8s further introduces an anchor-free design, enabling the model to directly learn object centers and bounding box coordinates without relying on predefined anchor boxes. This design reduces the constraints imposed by prior box configurations during training and improves both convergence stability and detection efficiency.

In the feature extraction process, the YOLOv8s detection head primarily relies on the local receptive fields of convolutional networks for feature aggregation, and performs object detection across different scales through multi-scale feature fusion. For brain tumor MRI images, lesion regions typically exhibit a wide range of scales and strong semantic saliency. Therefore, the local feature extraction capability of convolutional networks is well suited for lesion localization and boundary regression in this context.

In addition, the dense prediction paradigm adopted by YOLOv8s enables stable modeling over continuous spatial regions, making it well suited for large lesions with relatively continuous boundaries in medical images (23). Compared with multi-object detection tasks in complex natural scenes, brain tumor detection typically involves fewer targets and a relatively stable background structure. As a result, the convolution-based local feature modeling strategy of YOLOv8s provides certain advantages for this task. However, due to its reliance on local receptive fields, YOLOv8s exhibits inherent limitations in capturing long-range semantic dependencies. Whether the introduction of global modeling mechanisms can further improve brain tumor detection performance remains to be systematically validated through empirical experiments.

#### 1.1.4 Introduction and mechanism analysis of the RT-DETR detection head

Although YOLOv8s, based on a convolutional dense prediction mechanism, demonstrates certain advantages in object localization and training stability, its feature modeling process still relies primarily on local convolutional receptive fields, which limits its ability to capture long-range semantic dependencies and global contextual relationships. In recent years, with the rapid development of Transformer architectures in computer vision, object detection frameworks based on global attention mechanisms have attracted increasing research interest. Among them, RT-DETR (Real-Time Detection Transformer) introduces a query-based sparse prediction mechanism combined with a deformable attention module, enabling dynamic aggregation of global semantic information and achieving promising performance in complex object detection tasks (24).

Based on the above considerations, after completing hyper-parameter optimization for YOLOv8s, this study further introduces an RT-DETR detection head. Under identical training conditions, the original YOLOv8s detection head is replaced to investigate the applicability of a Transformer-based detection mechanism for brain tumor MRI detection tasks.

In contrast to the dense prediction-based detection paradigm of YOLOv8s, RT-DETR adopts a query-based sparse prediction mechanism, which models feature regions dynamically through a limited number of learnable queries. It further leverages deformable attention to perform adaptive feature aggregation and bounding box regression over target regions (25). Compared with traditional convolution-based detection frameworks, RT-DETR places greater emphasis on global semantic modeling and long-range feature dependencies. Although this design theoretically provides enhanced global representation capability, its actual effectiveness in specific application scenarios still requires empirical validation.

**Figure 3:**
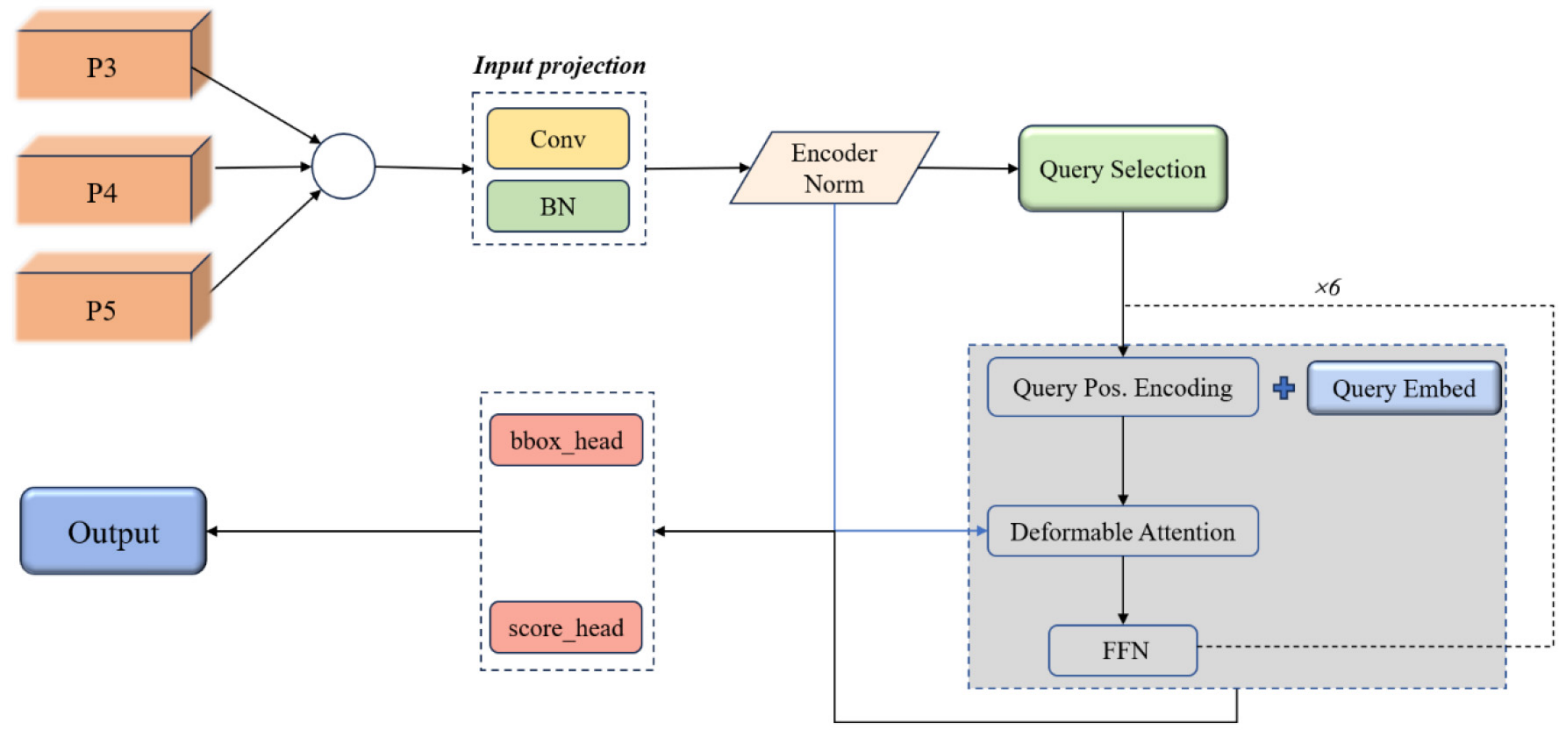
Schematic diagram of the RT-DETR detection head structure.

In addition, RT-DETR adopts a Transformer decoder architecture, which progressively refines target feature representations through multiple layers of attention mechanisms. It further incorporates a dynamic query selection strategy to enable adaptive focus on relevant target regions (26). This design has demonstrated strong global modeling capability in natural image object detection tasks, particularly in scenarios involving complex backgrounds, multiple objects, and dense object distributions. Therefore, this study further investigates the performance differences between CNN-based dense detectors and Transformer-based query-based detectors in brain tumor MRI detection tasks, with the aim of evaluating their respective adaptability to medical object detection scenarios. The structure of the RT-DETR detection head is illustrated in Figure 3.

## 2 Materials and methods

### 2.1 Overall technical workflow

**Figure 4:**
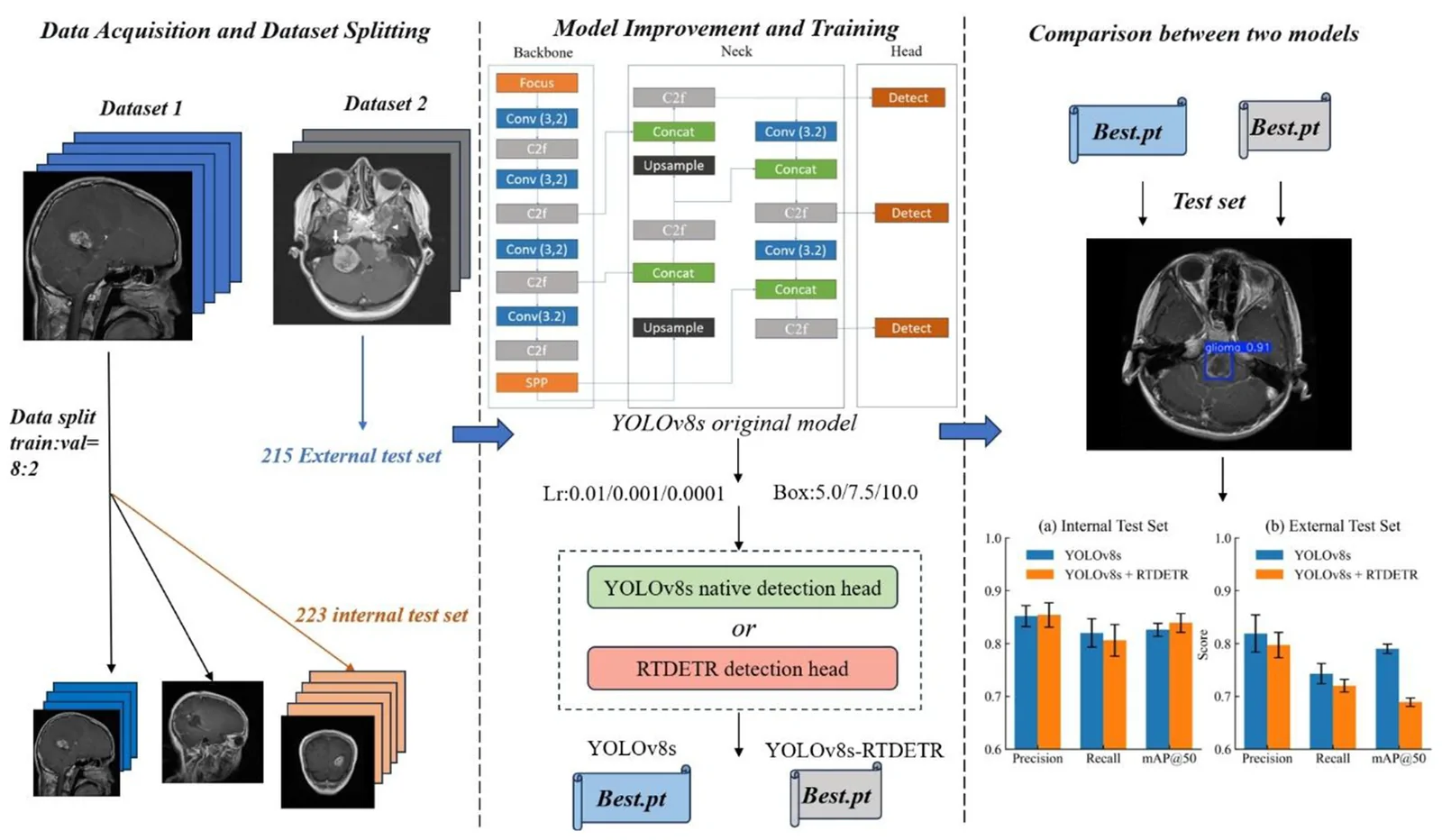
Technical workflow of this study.

The overall technical workflow of this study is illustrated in Figure 4. To comprehensively evaluate model effectiveness, the experimental design included baseline comparison, hyper-parameter ablation, detection-head ablation, and five-fold cross-validation for statistical robustness assessment.

First, two publicly available brain tumor MRI datasets were collected and subjected to standardized annotation procedures (27, 28). From Dataset 1, 3,000 images were randomly selected and split into training and validation sets with a ratio of 8:2, while the remaining 223 images were reserved as an internal test set for model training, parameter tuning, and preliminary performance evaluation. Dataset 2 was used as an external test set to assess the cross-domain generalization capability of the models.

Subsequently, a baseline model was constructed based on the YOLOv8s framework. A systematic hyper-parameter search was conducted by jointly varying the learning rate (0.01, 0.001, and 0.0001) and the bounding box regression loss weight (5.0, 7.5, and 10.0), resulting in nine experimental configurations. A five-fold cross-validation strategy was employed during training. The mean and standard deviation of each configuration were used to comprehensively evaluate detection performance and training stability, enabling the identification of an optimal hyper-parameter setting.

Based on the optimal hyper-parameter configuration, the YOLOv8s model was adopted as the baseline for further ablation experiments. Specifically, the original convolution-based decoupled detection head was replaced with a Transformer decoder-based detection head from RT-DETR. Comparative experiments were conducted under identical training conditions to investigate the differences in adaptability between the two detection mechanisms in brain tumor MRI detection tasks.

### 2.2 Data acquisition

The medical imaging data used in this study were obtained from two publicly available brain tumor MRI datasets on the Kaggle platform. The first dataset contains a total of 3,223 brain tumor MRI images, comprising two major intracranial tumor types: 1,581 cases of glioma and 1,642 cases of meningioma. This dataset, referred to as Dataset 1, was used for model training and internal testing. The second dataset includes 100 glioma cases and 115 meningioma cases, which was used as an external test set to evaluate model generalization performance.

The publicly available dataset lacked complete MRI acquisition metadata, including imaging parameters and protocols. Consequently, the potential impact of differences in imaging equipment and acquisition protocols between the external and internal datasets could not be assessed.

In this study, appropriate preprocessing steps were applied to the input images for YOLOv8s-based brain tumor MRI detection. These primarily included image resizing and pixel normalization. Specifically, all images with varying resolutions were uniformly resized to 640 × 640 pixels to meet the network input requirements. Subsequently, pixel intensities were linearly normalized from the range of 0–255 to 0, 1 to accelerate convergence and improve training stability.

All images were initially annotated using LabelImg by two board-certified radiologists with attending physician qualifications or higher. The annotations were further reviewed and validated *in situ* by two senior radiologists with associate chief physician qualifications or above to ensure annotation accuracy and experimental reliability.

**Figure 5:**
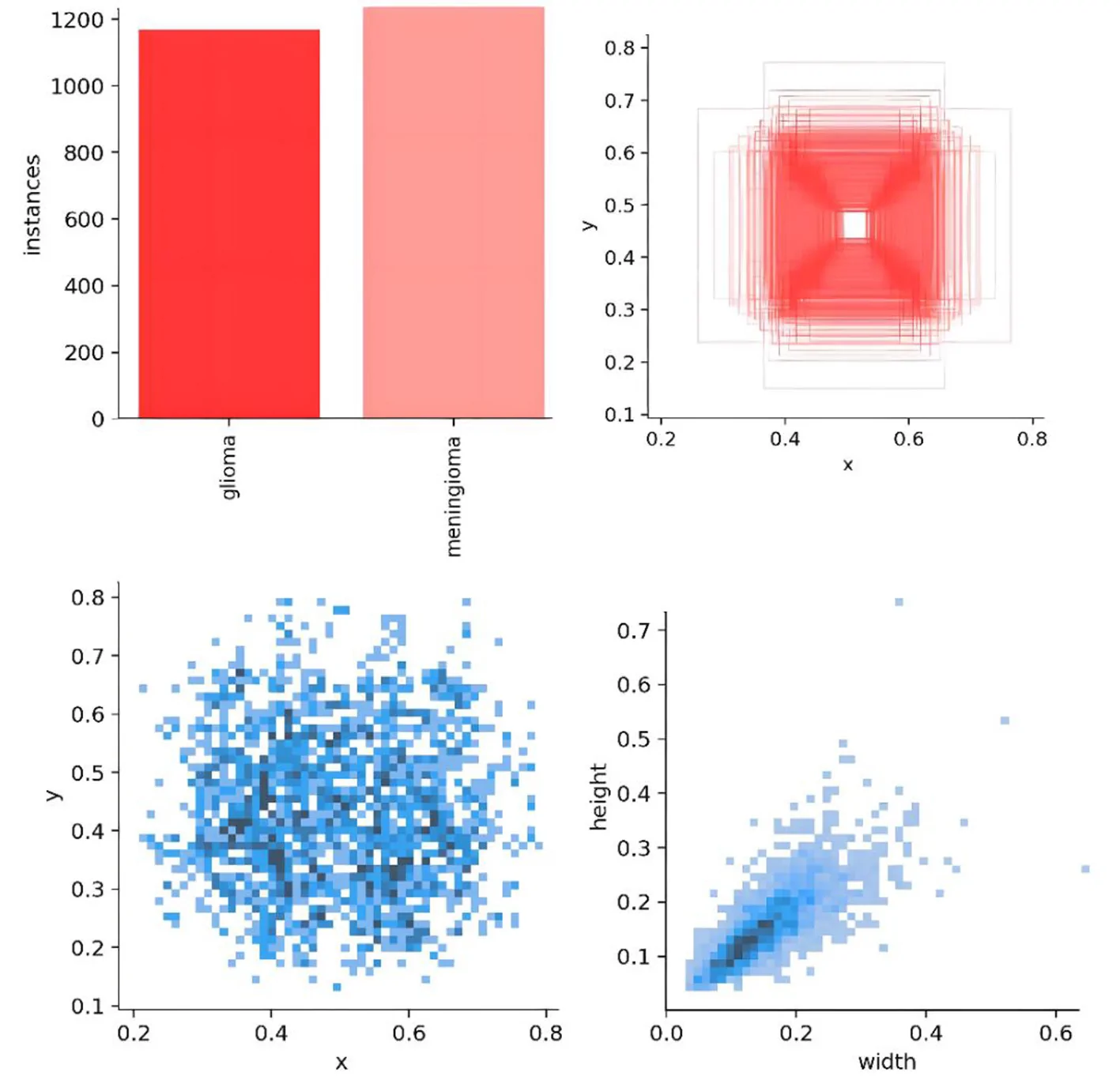
Visualization results of the training set images.

The model training process adopted a five-fold cross-validation strategy. From Dataset 1, 3,000 images were randomly selected and partitioned into five equal subsets, each containing 600 images. Each subset was then further split into training and validation sets with a ratio of 8:2. In each fold, four subsets were used for training and the remaining subset was used for validation, resulting in five rounds of rotation to construct training configurations with different data distributions. The remaining 223 independent test images were used to evaluate the object detection performance of the model. The distribution of tumor categories and bounding box annotations in the training set is visualized in Figure 5.

### 2.3 Experimental parameters

This study was conducted on a computing system equipped with a 13th-generation Intel(R) Core(TM) i7-13620H CPU running at 2.40 GHz, an NVIDIA GeForce RTX 4060 Laptop GPU, and 16 GB of RAM. The YOLOv8s model was implemented using the PyTorch framework (Python 3.8.7, PyTorch 2.0.0, CUDA 11.8).

For the nine hyperparameter configurations, all experiments were trained using the SGD optimizer across five datasets with different data distributions. The batch size was set to 8, and the number of training epochs was 100. During the validation and testing stages, an NMS IoU threshold of 0.7 was applied for predicted bounding box filtering. After identifying the optimal hyperparameter combination, the YOLOv8s detection head was replaced with an RT-DETR-based detection head. The modified model was then trained using five-fold cross-validation to obtain the final optimal weights.

### 2.4 Evaluation metrics

This study evaluates model performance using Precision, Recall, mAP@50, and GFLOPs. Precision is defined as the proportion of true positive (TP) predictions among all samples predicted as positive, reflecting the model's ability to avoid false positives (FP). A higher precision indicates fewer false positive detections. Recall is defined as the proportion of true positive cases correctly identified among all actual positive samples, reflecting the model's ability to detect all true lesions. A higher recall indicates fewer false negatives (FN). The corresponding formulations are given in Equations 4, 5, respectively.


$$\begin{array}{l} \begin{array}{c} Precision=TPTP+FP \end{array} \end{array}$$ (4)

$$\begin{array}{l} \begin{array}{c} Recall=TPTP+FN\; \end{array} \end{array}$$ (5)

mAP@50 refers to the mean Average Precision calculated at an Intersection over Union (IoU) threshold of 0.5. It evaluates the model's detection performance by measuring the average precision across recall levels when a predicted bounding box is considered correct if its IoU with the ground truth is greater than or equal to 0.5.

A higher mAP@50 indicates that the model can accurately detect target objects with better localization quality and fewer spatial deviations. It is a widely used metric for evaluating object detection performance, particularly in real-time applications where both accuracy and efficiency are critical.

The formulations of mAP and AP are given in Equations 6, 7, where p denotes precision, r denotes recall, and N represents the number of classes. Since this study is a binary classification task, the mAP value is equivalent to the AP value.


$$\begin{array}{l} \begin{array}{c} \overset{1}{\underset{0}{\int }}p\left(r\right)dr\;6 \end{array} \end{array}$$ (6)

$$\begin{array}{l} \begin{array}{c} mAP=N\;7 \end{array} \end{array}$$ (7)

## 3 Results analysis

Table 2 presents the five-fold cross-validation results of the YOLOv8s model under different combinations of learning rates and Box Loss weights for brain tumor MRI detection. Overall, the model performance is sensitive to both the learning rate and the Box Loss weight, and an interaction effect between these two hyperparameters can be observed to some extent.

**Table 2:** Five-fold cross-validation results under different combinations of learning rate and box Loss weight.

*Lr*	Box loss weight	*P*	*R*	mAP@50
0.01	5.0	0.916 ± 0.024	0.891 ± 0.011	0.926 ± 0.012
0.01	7.5	0.917 ± 0.017	0.892 ± 0.018	0.926 ± 0.015
0.01	10.0	0.910 ± 0.016	0.884 ± 0.013	0.925 ± 0.016
0.001	5.0	0.906 ± 0.027	0.895 ± 0.014	0.923 ± 0.021
0.001	7.5	0.921 ± 0.018	0.882 ± 0.021	0.926 ± 0.014
0.001	10.0	0.913 ± 0.011	0.890 ± 0.017	0.922 ± 0.013
0.0001	5.0	0.884 ± 0.019	0.862 ± 0.016	0.909 ± 0.019
0.0001	7.5	0.881 ± 0.037	0.859 ± 0.022	0.904 ± 0.019
0.0001	10.0	0.892 ± 0.005	0.854 ± 0.023	0.904 ± 0.017

From the perspective of learning rate, the overall detection performance shows a declining trend as the learning rate decreases from 0.01 to 0.0001. When the learning rate is set to 0.01, the model achieves consistently high performance across five folds, with mAP@50 ranging from 0.925 to 0.926, while both Precision and Recall remain at relatively high levels. In contrast, when the learning rate is reduced to 0.0001, mAP@50 decreases to 0.904–0.909, accompanied by a decline in Precision (0.881–0.892) and Recall (0.854–0.862). These results indicate that, for this task, a lower learning rate may lead to insufficient convergence efficiency, thereby degrading detection performance, whereas a relatively higher learning rate is more conducive to achieving better overall performance.

The impact of Box Loss weight on model performance is relatively limited, although certain consistent patterns can still be observed. Under a fixed learning rate, moderately increasing the Box Loss weight leads to slight improvements in both Precision and Recall. For example, when the learning rate is set to 0.01, increasing the Box Loss weight from 5.0 to 7.5 results in a marginal improvement in Precision from 0.916 to 0.917 and an increase in Recall from 0.891 to 0.892, while mAP@50 remains stable. These results suggest that a moderate strengthening of localization constraints may encourage the model to focus more on target regions; however, the overall performance gain is limited. When the Box Loss weight is further increased to 10.0, slight decreases in Precision, Recall, and mAP@50 are observed, indicating that overly strong localization constraints do not yield additional performance benefits and may, to some extent, disrupt the optimization balance among different learning objectives.

Overall, the learning rate exerts a more pronounced influence on model performance compared to the Box Loss weight, while the Box Loss weight primarily modulates the balance between localization precision and object recall. In this study, the combination of a learning rate of 0.01 and a Box Loss weight of 7.5 yields the most favorable overall performance in terms of Precision, Recall, and mAP@50. Therefore, this hyperparameter configuration is adopted as the baseline for all subsequent experiments.

Table 3 presents the five-fold cross-validation results of the optimal YOLOv8s detection head and the RT-DETR detection head for brain tumor MRI detection. Under identical training settings and data splits, the original YOLOv8s detection head consistently outperforms the RT-DETR detection head in terms of Precision, Recall, and mAP@50.

**Table 3:** Performance comparison between the optimal YOLOv8s detection head and the RT-DETR detection head.

Model	*P*	*R*	*mAP@50*
YOLOv8s (0.01+7.5)	0.917 ± 0.017	0.892 ± 0.018	0.926 ± 0.015
YOLOv8s + RT-DETR	0.913 ± 0.016	0.875 ± 0.023	0.911 ± 0.023
Difference	−0.004	−0.017	−0.015

Specifically, the optimal YOLOv8s model achieves a Precision of 0.917 ± 0.017, a Recall of 0.892 ± 0.018, and an mAP@50 of 0.926 ± 0.015. In contrast, after replacing the detection head with RT-DETR, Precision decreases to 0.913 ± 0.016, Recall decreases to 0.875 ± 0.023, and mAP@50 decreases to 0.911 ± 0.023. Among these metrics, Recall exhibits the most pronounced decline (−0.017), followed by mAP@50 (−0.015), while Precision shows only a marginal reduction (−0.004).

From the perspective of performance trends, the RT-DETR detection head has a relatively limited impact on Precision, indicating that the model can still maintain stable classification and localization performance for detected targets. However, a more noticeable decline is observed in Recall, suggesting a reduced ability to identify certain lesion regions. This further leads to a decrease in mAP@50, indicating that the overall detection performance of the RT-DETR detection head is inferior to that of the original YOLOv8s detection head in this task.

From the perspective of variability, both detection heads exhibit relatively small fluctuations across five-fold cross-validation, indicating overall training stability. Nevertheless, the RT-DETR detection head shows slightly higher standard deviations in Recall and mAP@50 compared to YOLOv8s, suggesting reduced robustness under different data splits.

**Figure 6:**
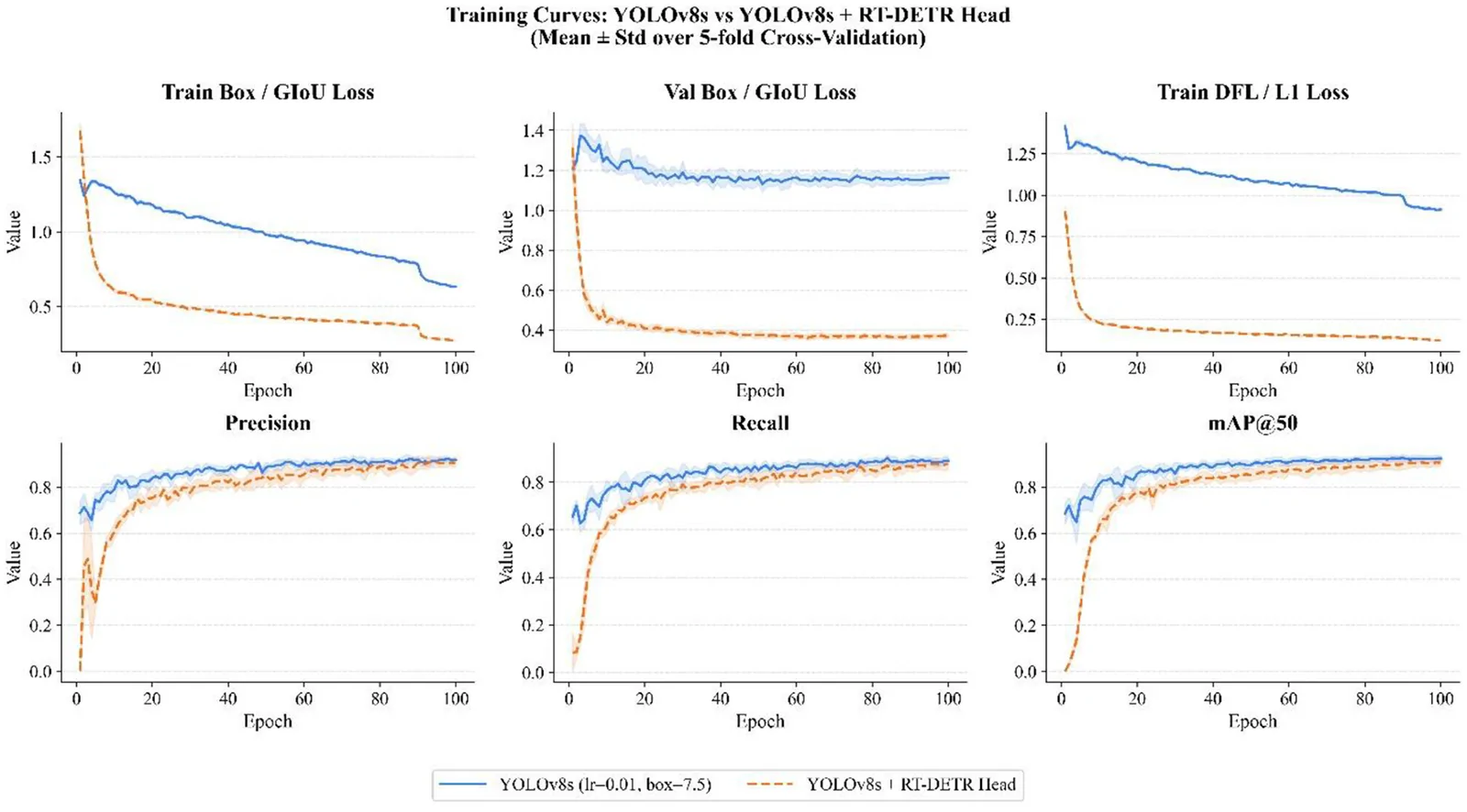
Training curves of the two models.

As shown in Figure 6, both the training and validation loss curves of all models exhibit stable convergence over 100 epochs, with no evidence of overfitting. Meanwhile, key evaluation metrics such as Precision, Recall, and mAP rapidly converge to a high and stable level after approximately 40 epochs, demonstrating strong training robustness and efficient convergence behavior.

From these results, the original YOLOv8s detection head achieves superior and more stable overall performance in brain tumor MRI detection tasks, whereas the RT-DETR detection head does not demonstrate clear advantages under the current experimental setting. Therefore, further discussion is warranted to investigate the applicability of different detection mechanisms with respect to task-specific characteristics.

To further examine performance differences under stricter localization requirements, additional evaluations were conducted at NMS IoU thresholds of 0.8 and 0.9 for both the YOLOv8s and RT-DETR detection heads, aiming to provide a more comprehensive assessment of their robustness in terms of localization accuracy.

**Figure 7:**
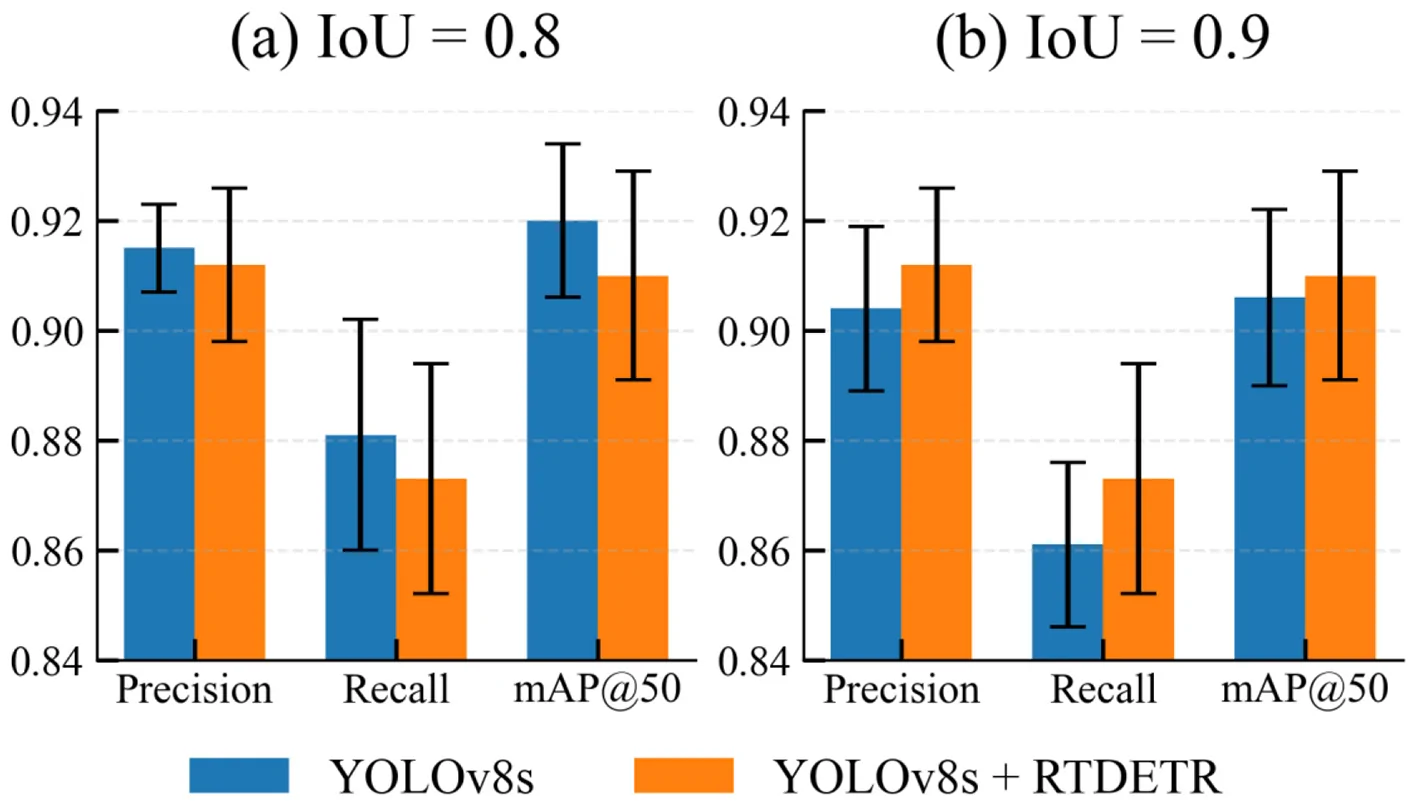
Bar chart of validation set evaluation metrics under different NMS IoU thresholds.

The experimental results are presented in Table 4 and the corresponding visualization in Figure 7. At an NMS IoU threshold of 0.9 the evaluation results are summarized in Table 5, the RT-DETR head exhibited a substantially smaller performance decline than the native YOLOv8s head. Specifically, the mAP@50 of YOLOv8s decreased by approximately 0.020 as the NMS IoU threshold increased from 0.7 to 0.9, whereas RT-DETR decreased by only 0.001, indicating greater robustness to NMS threshold variations. This may be attributed to the sparse query mechanism of RT-DETR, which generates fewer and less-overlapping candidate boxes, making its predictions less sensitive to NMS. In contrast, the dense prediction strategy of YOLOv8s produces more redundant predictions, which may increase false positives under higher NMS thresholds and consequently reduce mAP.

**Table 4:** Model evaluation metrics at NMS IoU = 0.8.

Model	Precision	Recall	mAP@50
YOLOv8s (0.01+7.5)	0.915 ± 0.008	0.881 ± 0.021	0.920 ± 0.014
YOLOv8s + RT-DETR	0.912 ± 0.014	0.873 ± 0.021	0.910 ± 0.019

**Table 5:** Model evaluation metrics at NMS IoU = 0.9.

Model	Precision	Recall	mAP@50
YOLOv8s (0.01+7.5)	0.904 ± 0.015	0.861 ± 0.015	0.906 ± 0.016
YOLOv8s + RT-DETR	0.912 ± 0.014	0.873 ± 0.021	0.910 ± 0.019

These findings suggest that the two detection mechanisms exhibit distinct performance degradation patterns under increasing NMS IoU constraints, which will be further analyzed in the Discussion section.

**Figure 8:**
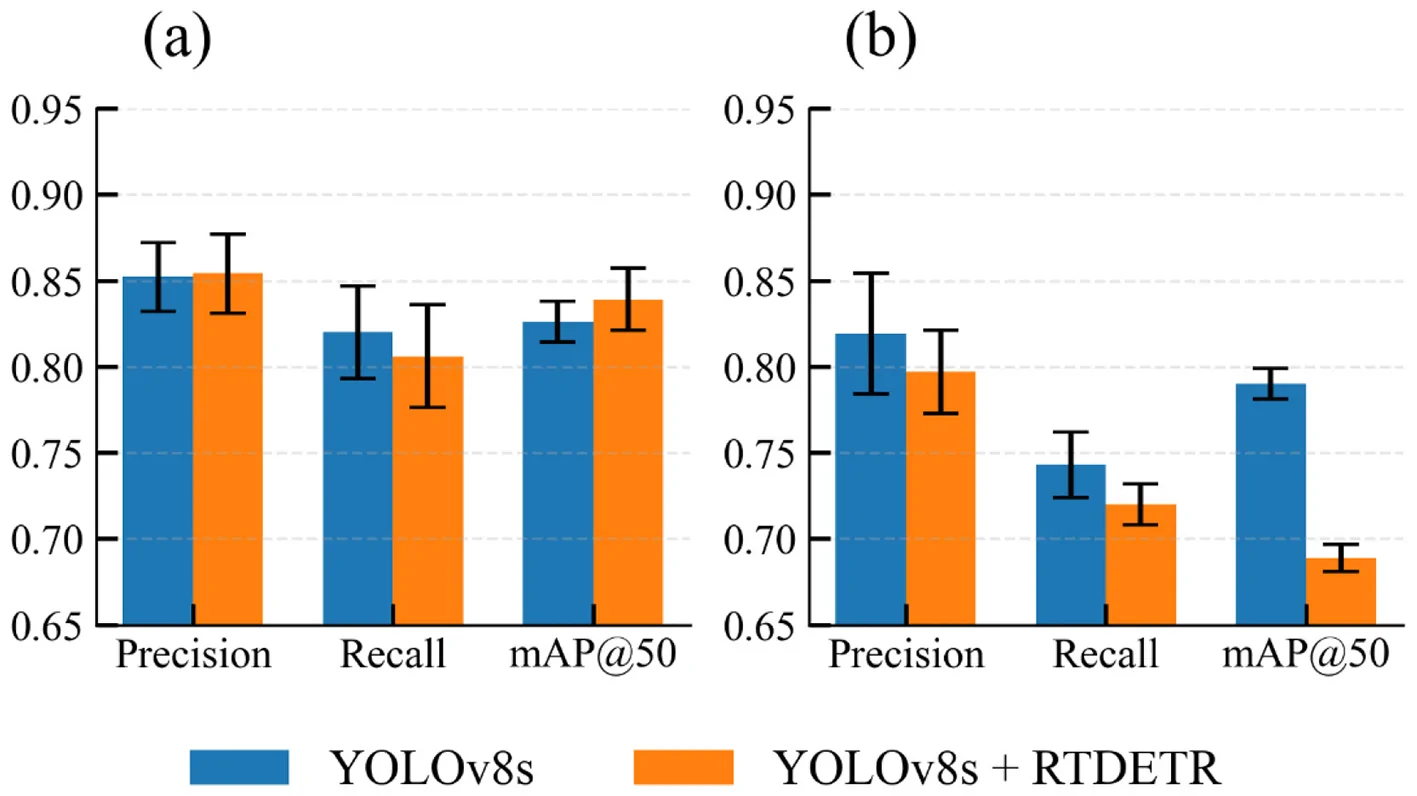
Bar chart of results on the **(a)** internal and **(b)** external test sets.

To further evaluate the generalization capability of the two detection mechanisms on unseen data, this study utilized the five optimal weight checkpoints obtained from five-fold cross-validation. These models were separately evaluated on an internal test set (223 images) and an external test set (215 images). The final performance metrics were reported as the mean and standard deviation of the five-fold inference results to comprehensively reflect model generalization stability. The evaluation results on the internal and external test sets are presented in Table 6 and Table 7, respectively, while the corresponding quantitative results are visualized in Figure 8.

**Table 6:** Model evaluation metrics on the internal test set.

Model	Precision	Recall	mAP@50
YOLOv8s (0.01+7.5)	0.852 ± 0.020	0.820 ± 0.027	0.826 ± 0.012
YOLOv8s + RT-DETR	0.854 ± 0.023	0.806 ± 0.030	0.839 ± 0.018

**Table 7:** Model evaluation metrics on the external test set.

Model	Precision	Recall	mAP@50
YOLOv8s (0.01+7.5)	0.819 ± 0.035	0.743 ± 0.019	0.790 ± 0.009
YOLOv8s + RT-DETR	0.797 ± 0.024	0.720 ± 0.012	0.689 ± 0.008

On the internal test set, the YOLOv8s+RT-DETR model achieves slightly higher Precision (0.854 ± 0.023) and mAP@50 (0.839 ± 0.018) compared with the original YOLOv8s model (0.852 ± 0.020 and 0.826 ± 0.012, respectively), while its Recall (0.806 ± 0.030) is lower than that of the baseline model (0.820 ± 0.027). Overall, the performance differences between the two models are minimal, indicating comparable performance levels.

On the external test set, both models exhibit varying degrees of performance degradation, reflecting the challenges associated with cross-domain generalization. The original YOLOv8s model consistently outperforms the YOLOv8s+RT-DETR variant across all metrics, achieving a Precision of 0.819 ± 0.035, a Recall of 0.743 ± 0.019, and an mAP@50 of 0.790 ± 0.009. In comparison, the YOLOv8s+RT-DETR model yields lower results (Precision: 0.797 ± 0.024, Recall: 0.720 ± 0.012, mAP@50: 0.689 ± 0.008), with the most pronounced difference observed in mAP@50, which decreases by 0.101.

**Figure 9:**
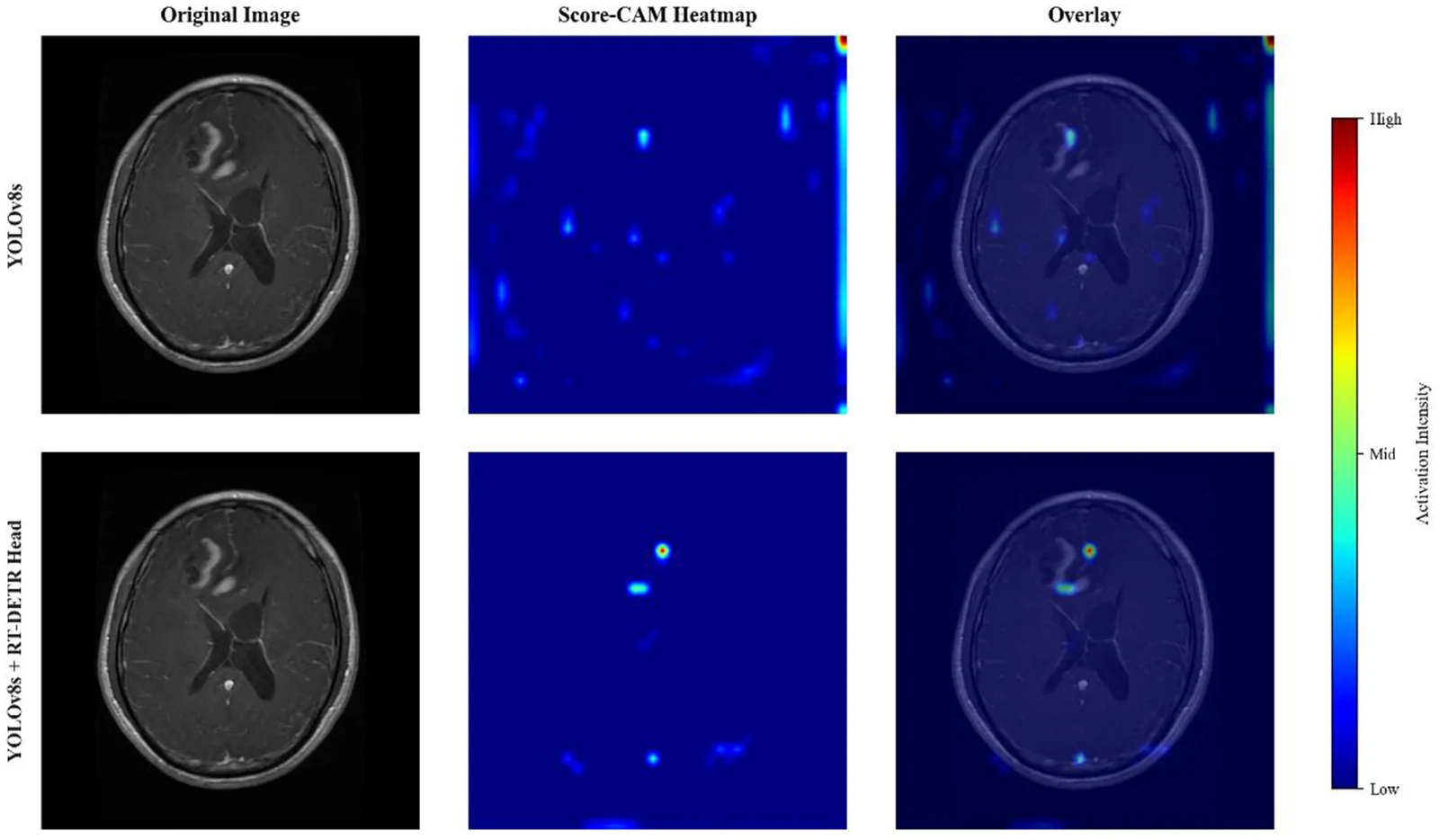
Score-CAM visualization heatmaps on the internal test set.

**Figure 10:**
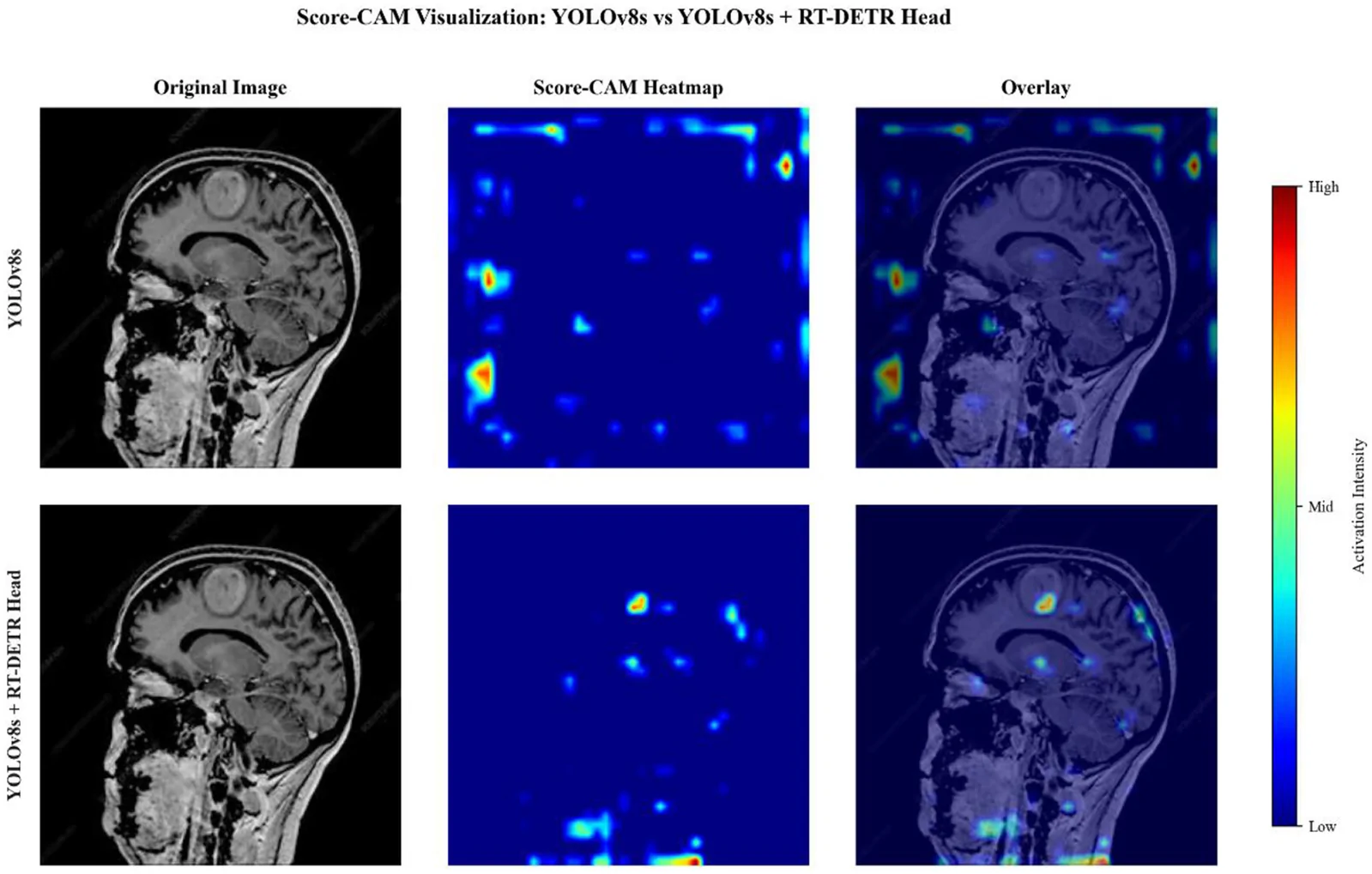
Score-CAM visualization heat maps on the external test set.

To facilitate a fair comparison between the native YOLOv8 and Transformer-based RT-DETR detection heads, a gradient-free visualization method was employed. Unlike gradient-based CAM methods, Score-CAM estimates feature importance via forward perturbation rather than back-propagated gradients, reducing architecture-specific bias and providing a more consistent basis for qualitative comparison between heterogeneous detection heads. The results are shown in Figures 9, 10. It can be observed that both detection heads generate strong activation responses in tumor regions, indicating that both models successfully learn discriminative features associated with lesion areas. However, the two detection heads exhibit notable differences in their activation patterns, and a more detailed analysis will be provided in the Discussion section.

## 4 Discussion

Despite the high detection or classification performance achieved by existing deep learning studies on brain tumors, most models are optimized for isolated tasks, with limited systematic evaluation of detection mechanisms and training strategies. To address this gap, this study leverages the YOLOv8s framework to comprehensively assess brain tumor MRI detection performance across multiple dimensions, including training parameters, detection architectures, inference configurations, and cross-domain generalization. Specifically, we investigate parameter sensitivity via learning rate and bounding box regression loss weight, compare the convolutional dense prediction and Transformer sparse query mechanisms by substituting the RT-DETR detection head, and evaluate model stability under varying NMS IoU thresholds and external test sets. Prior work has established that training strategy optimization—encompassing data augmentation, optimizer selection, and hyperparameter tuning—plays a critical role in enhancing deep learning performance in medical imaging. Experimental results indicate that the learning rate is the dominant hyperparameter influencing model performance, while the effect of the Box Loss weight is relatively moderate within a reasonable range. Moreover, a clear interaction effect between these two hyperparameters is observed. In the detection head comparison, the native YOLOv8s detection head demonstrated superior overall detection performance and cross-domain generalization, whereas the RT-DETR detection head maintained more stable performance across different NMS IoU thresholds, exhibiting greater robustness to post-processing. These findings suggest that the two detection mechanisms exhibit distinct and complementary performance characteristics. The following discussion further interprets these results in light of existing literature.

As one of the most critical hyperparameters in deep learning optimization, the learning rate directly determines the magnitude of parameter updates and the convergence behavior of the model, and it serves as the dominant factor influencing YOLOv8s performance in brain tumor detection, with a substantially greater impact than the Box Loss weight. In this study, when the learning rate decreases from 0.01 to 0.0001, a significant decline in mAP@50 is observed, with an average reduction of approximately 0.020. Meanwhile, training stability also deteriorates, with the standard deviation reaching up to 0.037 in certain configurations. This observation is consistent with gradient descent optimization theory: when the learning rate is too small, parameter updates become insufficient, preventing the model from adequately minimizing the loss function within a limited number of training epochs, and causing it to converge to suboptimal local minima, thereby degrading generalization performance. These findings are consistent with previous studies reporting the critical role of learning rate in object detection performance (29, 30), further confirming the importance of proper learning rate selection in medical image detection tasks.

Building on the identification of the learning rate as the dominant factor, the role of the Box Loss weight in modulating model performance is further analyzed. The results indicate that, within a reasonable learning rate range, the Box Loss weight exerts a relatively mild influence on performance, with mAP@50 variations among different configurations remaining within 0.004. This suggests that YOLOv8s exhibits a certain degree of robustness to this hyperparameter. This behavior is closely related to the design of the multi-task loss function in YOLOv8s. Whenλ_*box*_is excessively large, the contribution of the bounding box regression loss to the overall gradient becomes dominant, potentially suppressing the optimization of the classification loss $L_{cls}\;$and distribution focal loss $L_{dfl}$, thereby reducing the learning efficiency of classification confidence. This finding is consistent with previous work by Zhang et al. (31) on the impact of multi-task loss weighting on detection performance.

After completing the hyperparameter optimization experiments and determining the baseline configuration, this study further investigates the adaptability of different detection heads in brain tumor MRI detection from a detection-mechanism perspective. Previous studies have demonstrated the feasibility of applying YOLO-based object detection models to brain tumor MRI detection. By fine-tuning YOLOv10 through transfer learning, high detection accuracy was achieved under limited training data, highlighting the suitability of one-stage detectors for medical imaging. In contrast, while that study focused on transfer learning strategies, the present study investigates the adaptability of different detection mechanisms for brain tumor MRI detection (32). The original YOLOv8s detection head adopts a convolutional neural network–based dense prediction paradigm, performing joint regression of object categories and bounding boxes across multi-scale feature maps (33). Convolutional neural networks extract spatial features through local receptive fields and weight sharing, thereby introducing a strong local inductive bias that enables effective feature learning even under limited data conditions (34). In addition, YOLOv8s incorporates a decoupled detection head and an anchor-free strategy, which separates classification and regression tasks, reduces inter-task interference, and improves training stability as well as adaptability to objects at different scales.

In contrast, the RT-DETR detection head adopts a Transformer decoder–based sparse prediction paradigm, where object detection is formulated through a limited set of object queries (35), and multi-scale feature aggregation is achieved via deformable attention. The Transformer architecture leverages self-attention to establish global feature interactions, thereby providing stronger capability in modeling long-range dependencies compared with convolutional structures. Meanwhile, RT-DETR iteratively refines object queries through multi-layer decoders and incorporates global contextual information to progressively improve prediction quality, leading to enhanced bounding box localization accuracy (36). This observation is consistent with findings from brain tumor MRI segmentation studies. By incorporating a Transformer module into ResNet U-Net, TransResUNet effectively combines local feature extraction with global contextual modeling, achieving superior segmentation performance on the BraTS2019 dataset. These findings suggest that Transformer architectures can effectively enhance long-range dependency modeling in medical images (37).

However, the global modeling capability of Transformers is generally more dependent on large-scale training data, and the object query optimization process also requires sufficient data diversity to ensure stable convergence (38, 39). Therefore, in medical imaging scenarios with limited sample sizes, its performance is more susceptible to variations in data distribution, which may consequently lead to reduced model stability.

As the NMS IoU threshold increased from 0.7 to 0.9, the two detection paradigms exhibited distinct performance trends. The mAP@50 of the native YOLOv8s detection head gradually declined with increasing NMS IoU thresholds, whereas the RT-DETR head maintained relatively stable performance. This difference cannot be attributed to changes in the evaluation criterion, as all experiments were evaluated using a fixed mAP@50 metric. Instead, it primarily reflects differences in candidate box generation and sensitivity to NMS post-processing between the two detection mechanisms.

Specifically, the YOLOv8s detection head adopts a dense prediction strategy that generates a large number of candidate boxes across multi-scale feature maps, improving lesion coverage. However, for brain tumor MRI lesions with indistinct boundaries or complex morphology, dense prediction may produce multiple highly overlapping candidates. At higher NMS IoU thresholds, more redundant predictions are retained, potentially increasing duplicate detections and false positives, thereby reducing precision and overall detection performance.

In contrast, RT-DETR employs a sparse query-based prediction mechanism that optimizes a limited set of object queries through the Transformer decoder, substantially reducing redundant candidate boxes. Consequently, its predictions are less sensitive to changes in the NMS IoU threshold, resulting in more stable detection performance across different threshold settings.

Overall, the NMS IoU sensitivity analysis demonstrates that the YOLOv8s and RT-DETR detection heads exhibit distinct post-processing characteristics during inference. While the dense prediction strategy provides stronger candidate coverage, it is more sensitive to NMS settings. In contrast, the sparse query mechanism reduces redundant predictions and exhibits greater robustness to variations in NMS parameters. These findings highlight the inherent differences between dense prediction and sparse query mechanisms in brain tumor MRI detection, rather than indicating a general superiority of either detection paradigm. Therefore, the performance variations under different NMS IoU conditions further indicate that relying solely on a single evaluation threshold may be insufficient to comprehensively reflect the actual behavioral characteristics of detection models. For medical object detection tasks, models are required not only to achieve high detection accuracy but also to maintain stable performance under varying inference strategies.

In the independent test set evaluation of generalization performance, both detection heads exhibit varying degrees of performance degradation, with RT-DETR showing a more pronounced decline. Although both the training set and external test set consist of brain tumor MRI data, differences across imaging centers—such as scanner hardware, magnetic field strength, imaging protocols, reconstruction algorithms, and patient population characteristics—can introduce significant variations in intensity distribution, tissue contrast, and lesion boundary appearance. When a distribution shift exists between the feature space learned during training and the input data at test time, both feature representation and decision boundaries of the model may be adversely affected, leading to degraded detection performance.

The more substantial performance deterioration observed in RT-DETR can be attributed to its reliance on object queries and dynamic attention mechanisms for target representation learning. When the input feature distribution shifts, the learned attention relationships may become misaligned, thereby weakening the model's ability to represent lesion regions effectively. In addition, Transformer-based architectures lack the strong local inductive bias inherent in convolutional neural networks, and their performance is generally more dependent on large-scale training data and consistent data distributions to learn stable attention patterns. In medical imaging scenarios with limited training samples, this property may further amplify the model's sensitivity to distribution shifts, resulting in increased performance variability across different datasets.

In contrast, the original YOLOv8s detection head relies on local receptive fields and weight-sharing mechanisms to extract low-level visual features such as edges and textures. These features are generally more robust to variations in imaging conditions, which enables more stable performance in cross-domain testing. These findings suggest that, under the current dataset scale for brain tumor MRI detection, convolution-based detectors still demonstrate stronger generalization capability, whereas Transformer-based detectors may require larger-scale training data, pretraining strategies, or domain adaptation methods to fully exploit their global modeling capacity.

To further validate the above quantitative findings from an interpretability perspective, Score-CAM was employed to visualize feature activation distributions of both detection heads across multiple samples. The results show that the YOLOv8s detection head produces relatively widespread activation maps, with responses not only in tumor core regions but also in brain tissue boundaries, periventricular areas, and parts of normal tissue. This pattern reflects the typical dense prediction behavior of convolutional neural networks. Such broad activation is consistent with its higher recall performance: extensive feature attention helps capture tumor-related contextual information and reduces the risk of missed detections, although it may also introduce background-related redundancy.

The heatmaps of the RT-DETR detection head exhibited a distinctly different attention pattern, with high-activation regions concentrated on the tumor lesions and their surrounding areas, while responses in the background were substantially suppressed. This suggests that the sparse query mechanism provides more selective attention to clinically relevant regions. These attention patterns are consistent with the findings of the NMS IoU sensitivity analysis: RT-DETR maintained more stable detection performance across different NMS IoU thresholds, whereas YOLOv8s was more sensitive to variations in NMS settings. Together, these results indicate inherent differences in candidate box generation and post-processing robustness between sparse query and dense prediction mechanisms, providing interpretability-based evidence for selecting appropriate detection strategies according to different clinical requirements.

A unified Score-CAM framework was adopted to ensure a consistent interpretability setting for comparing different detection mechanisms, thereby minimizing the influence of architectural differences on the visualization results. The interpretability analysis was intended to qualitatively compare attention patterns between models rather than evaluate different explainability methods. Future studies may incorporate additional CAM-based visualization techniques to cross-validate model attention regions and further improve the reliability of the interpretability analysis.

Overall, the present study revealed a clear performance trade-off between convolution-based and Transformer-based detection heads for brain tumor MRI detection. Benefiting from its dense prediction strategy and the inherent local inductive bias of convolutional networks, the native YOLOv8s detection head demonstrated superior lesion detection capability, training stability, and cross-domain generalization. In contrast, the RT-DETR detection head, leveraging the Transformer's global modeling capability and sparse query mechanism, maintained more stable detection performance across different NMS IoU thresholds, exhibiting greater robustness to post-processing. Therefore, the native YOLOv8s detection head may be better suited for clinical screening scenarios that prioritize lesion detection efficiency, whereas the RT-DETR detection head may be more advantageous for applications requiring higher detection stability and model robustness.

Several limitations of this study should be acknowledged. First, both datasets were obtained from the publicly available Kaggle platform and were relatively limited in scale. In addition, the lack of clinical information, such as patient age and tumor grade, precluded a more comprehensive evaluation of model generalizability across disease subtypes. Furthermore, the external dataset lacked complete MRI acquisition metadata, preventing assessment of potential domain shifts arising from differences in imaging equipment and acquisition protocols. Future work will incorporate multicenter MRI datasets with complete acquisition parameters to further validate model generalizability and domain adaptability.

Second, the hyperparameter analysis was limited to the learning rate and bounding box regression loss weight, whereas other influential factors, including data augmentation strategies and weight decay, were not systematically investigated. Moreover, the RT-DETR detection head was evaluated only through direct replacement without task-specific architectural optimization. Therefore, the current findings may not fully reflect the potential of Transformer-based detection mechanisms for brain tumor MRI detection.

Future research may focus on two key directions. Beyond improving detection accuracy, greater emphasis should be placed on enhancing model interpretability and clinical transparency. Recent studies have incorporated explainable artificial intelligence into brain tumor MRI analysis. For example, EASE-Net combines ensemble learning with LIME to provide case-level visual explanations, while CNN-TumorNet also employs LIME to improve the interpretability of classification decisions, highlighting the growing importance of explainability for clinically deployable AI systems (40, 41). Future work should further explore the integration of explainability techniques with object detection models to improve clinician trust and facilitate clinical translation.

Future studies should focus on developing unified frameworks capable of performing multiple clinical tasks simultaneously. Previous research has demonstrated the feasibility of jointly optimizing brain tumor segmentation and classification within a multitask learning framework (42). Extending this paradigm to object detection could promote feature sharing across related tasks and enable more comprehensive lesion characterization. Furthermore, hybrid detection frameworks that combine multiple detection mechanisms may offer additional advantages. For example, dual-branch feature fusion or lightweight attention modules could be incorporated to enhance global contextual modeling while preserving the real-time efficiency of the YOLO architecture, thereby improving the detection of small lesions and indistinct tumor boundaries in complex medical images. Previous studies have shown that CNN–Transformer hybrid architectures achieve superior generalization and stability in medical imaging tasks (43), providing valuable guidance for future model design. Collectively, these advances are expected to facilitate the development of more robust, interpretable, and clinically applicable brain tumor analysis systems.

From a clinical application perspective, YOLOv8s, as a single-stage detection framework, offers high inference efficiency and low computational complexity, making it more suitable for rapid imaging screening scenarios compared to methods that rely on complex post-processing pipelines. Future work incorporating lightweight deployment, model compression, and multi-center data validation holds promise for further translating this approach into clinical auxiliary diagnostic systems.

## 5 Conclusion

This study is based on the YOLOv8s framework and systematically analyzes the impact of the learning rate and bounding box regression loss weight on brain tumor MRI detection performance. Furthermore, a comparative evaluation is conducted between convolution-based and Transformer-based detection heads to assess their adaptability to this task. The results indicate that the optimal configuration in this study is achieved with a learning rate of 0.01 and a Box Loss Weight of 7.5, yielding the best overall performance with a five-fold cross-validation AP@0.5 of 0.926 ± 0.015.

The detection head comparison demonstrated that the native YOLOv8s detection head achieved superior recall and generalization performance on conventional detection metrics and the external test set. In contrast, the RT-DETR detection head maintained more stable performance across different NMS IoU thresholds, indicating greater robustness to variations in inference-stage post-processing. These findings highlight the complementary characteristics of the two detection paradigms: the dense prediction mechanism provides stronger lesion coverage, whereas the sparse query mechanism offers greater prediction stability.

Overall, under the current dataset scale and application setting, the native YOLOv8s detection head appears more suitable for brain tumor MRI–based computer-aided screening, while the RT-DETR detection head shows greater potential for reducing redundant predictions and improving inference stability. The findings of this study provide practical guidance for hyperparameter optimization, detection head selection, and inference strategy design in medical object detection tasks.

## Data Availability

Publicly available datasets were analyzed in this study. This data can be found here: https://www.kaggle.com/datasets/masoudnickparvar/brain-tumor-mri-dataset~https://www.kaggle.com/datasets/sartajbhuvaji/brain-tumor-classification-mri.

## References

[1] Miller K Ostrom Q Kruchko C Patil N Tihan T Cioffi G . Brain and other central nervous system tumor statistics, 2021. CA Cancer J Clin. (2021) 71:381–406. doi: 10.3322/caac.21693 34427324

[2] GBD 2016 Brain and Other CNS Cancer Collaborators. Global, regional, and national burden of brain and other CNS cancer, 1990–2016: a systematic analysis for the Global Burden of Disease Study 2016. Lancet Neurol. (2019) 18:376–93. doi: 10.1016/S1474-4422(18)30468-X 30797715 PMC6416167

[3] Chong AW McAdory LE Low DC . Primary intraventricular tumors - Imaging characteristics, post-treatment changes and relapses. Clin Imaging. (2022) 82:38–52. doi: 10.1016/j.clinimag.2021.10.008 34773811

[4] Saeedi S Rezayi S Keshavarz H Kalhori SNR. MRI-based brain tumor detection using convolutional deep learning methods and chosen machine learning techniques. BMC Med Inform Decis Mak. (2023) 23:16. doi: 10.1186/s12911-023-02114-6 36691030 PMC9872362

[5] Burhan A Purwono P. A comprehensive review of knowledge distillation for lightweight medical image segmentation. J Adv Health Inform Res. (2024) 2:95–101. doi: 10.59247/jahir.v2i2.294

[6] Redmon J Divvala S Girshick R Farhadi A. You only look once: unified, real-time object detection. In: Proceedings of the IEEE Conference on Computer Vision and Pattern Recognition (CVPR). Las Vegas, NV: IEEE (2016). p. 779–88. doi: 10.1109/CVPR.2016.91

[7] Chen F Zhang Y Fu L Xie H Zhang Q Bi S. From YOLOv5 to YOLOv8: structural innovations and performance improvements. Int J Comput Sci Inf Technol. (2025) 6:93. doi: 10.62051/ijcsit.v6n1.12.

[8] Cao J Zhang T Hou L Nan N. An improved YOLOv8 algorithm for small object detection in autonomous driving. J Real-Time Image Process. (2024) 21:98. doi: 10.1007/s11554-024-01517-6

[9] Tuteja P Arora S Bhardwaj A Wani NA Ahmad N Alshara MA . Augmented multimodal fusion for optimized brain tumor detection: evaluation and comparative analysis. JoVE. (2025) p. 220. doi: 10.3791/67822 40587439

[10] Atamna A Maus T Kievelitz F Glasmachers T. Cumulative learning rate adaptation: revisiting path-based schedules for SGD and Adam. arXiv [Preprint] arXiv:2508.05408. (2025).

[11] Macêdo D Zanchettin C Ludermir T. Sigmoidal learning rate optimizer for deep neural network training using a two-phase adaptation approach. Appl Soft Comput. (2024) 167:112264. doi: 10.1016/j.asoc.2024.112264

[12] Deepak G Bhat S. Optimization of deep learning-based faster R-CNN network for vehicle detection. Sci Rep. (2025) 15:8142. doi: 10.1038/s41598-025-22828-z 41198791 PMC12592562

[13] Liu C Wang K Li Q Zhao F Zhao K. Powerful-IoU: more straightforward and faster bounding box regression loss with a nonmonotonic focusing mechanism. Neural Netw. (2023) 170:276–84. doi: 10.1016/j.neunet.2023.11.041 38000311

[14] Wu S Yang J Wang X Li X. IoU-Balanced loss functions for single-stage object detection. Pattern Recognit Lett. (2022) 4:156.

[15] F. I G S R, editors. Deep Learning on Brain MRI: ResNet50V2 vs. DenseNet121 Meningioma Detection. In: 4th International Conference on Innovative Mechanisms for Industry Applications (ICIMIA) (2025).

[16] Rajendran S Rajagopal SK Thanarajan T Shankar K Kumar S Alsubaie NM . Automated Segmentation of Brain Tumor MRI Images Using Deep Learning. IEEE Access. (2023) 11:64758–68. doi: 10.1109/ACCESS.2023.3288017

[17] Jemina SL Thanarajan T. An intelligent brain tumor detection model using lightweight hybrid twin attentive pyramid convolutional network. Sci Rep. (2025) 15:40177. doi: 10.1038/s41598-025-23813-2 41249242 PMC12624066

[18] Jemina SL T T S. Brain Tumor Detection Using Extended Optimization Based Multi-Layer Perceptron. In:W B, editors. Proceedings in International Conference on Computing Technologies & Data Communication (ICCTDC). (2025). doi: 10.1109/ICCTDC64446.2025.11158990

[19] Ren Z Yao K Sheng S Wang B Lang X Wan D . YOLO-SDH: improved Y0OLOv5 using scaled decoupled head for object detection. Int J Mach Learn Cybern. (2024) 16:1643–60. doi: 10.1007/s13042-024-02357-3

[20] Tong Y Ye H Yang J Yang X. ACD-DETR. Adaptive cross-scale detection transformer for small object detection in UAV imagery. Sensors. (2025) 25:5556. doi: 10.3390/s25175556 40942984 PMC12431001

[21] Wang C Gong X. Bounding box regression with balance for harmonious object detection. J Vis Commun Image Represent. (2022) 89:103665. doi: 10.1016/j.jvcir.2022.103665

[22] Vasanthi P Mohan L. Efficient YOLOv8 algorithm for extreme small-scale object detection. Digit Signal Process. (2024) 154:104682. doi: 10.1016/j.dsp.2024.104682

[23] Yao Q Zhuang D Feng Y Wang Y Liu J. Accurate detection of brain tumor lesions from medical images based on improved YOLOv8 algorithm. IEEE Access. (2024) 12:144260–79. doi: 10.1109/ACCESS.2024.3472039

[24] Zhu X Su W Lu L Li B Wang X Dai J. Deformable DETR: deformable transformers for end-to-end object detection. arXiv [Preprint] arXiv:2010.04159. (2020).

[25] Wang S Xia C Lv F Shi Y. RT-DETRv3: real-time end-to-end object detection with hierarchical dense positive supervision. In: Proceedings of the IEEE/CVF Winter Conference on Applications of Computer Vision (WACV). (2025). p. 1628–36. doi: 10.1109/wacv61041.2025.00166

[26] Dai X Chen Y Yang J Zhang P Yuan L Zhang L. Dynamic DETR: end-to-end object detection with dynamic attention. In: Proceedings of the IEEE/CVF International Conference on Computer Vision (ICCV). (2021). p. 2968–77. doi: 10.1109/ICCV48922.2021.00298

[27] Nickparvar M. Brain tumor MRI dataset [Internet]. Kaggle; (2021. Available online at: https://www.kaggle.com/datasets/masoudnickparvar/brain-tumor-mri-dataset (Accessed February 18 2026).

[28] Bhuvaji S Kadam A Dedge S Kanchan S. Brain tumor classification (MRI) [Internet]. Kaggle (2020). Available online at: https://www.kaggle.com/datasets/sartajbhuvaji/brain-tumor-classification-mri (Accessed February 18 2026).

[29] Bisla D Wang J Choromańska A. Low-pass filtering SGD for recovering flat optima in the deep learning optimization landscape. arXiv [Preprint] arXiv:2201.08025. (2022).

[30] Chen S Zhang C Mu H. An adaptive learning rate deep learning optimizer using long and short-term gradients based on G-L fractional-order derivative. Neural Process Lett. (2024) 56:48. doi: 10.1007/s11063-024-11571-7

[31] Zhang YF Ren W Zhang Z Jia Z Wang L Tan T. Focal and efficient IoU loss for accurate bounding box regression. arXiv [Preprint] arXiv:2101.08158. (2021).

[32] Chhimpa GR Awasthi S Bhati N Yadav P Wani NA. A transfer learning-driven fine-tuning of YOLOv10 for improved brain tumor detection in MRI images. Scientific Reports. (2025). 16. 10.1038/s41598-025-28813-w PMC12764844 41326486

[33] Ge Z Liu S Wang F Li Z Sun J. YOLOX: exceeding YOLO series in 2021. arXiv [Preprint] arXiv:2107.08430. (2021).

[34] LeCun Y Bengio Y Hinton G. Deep learning. Nature. (2015) 521:436–44. doi: 10.1038/nature14539 26017442

[35] Carion N Massa F Synnaeve G Usunier N Kirillov A Zagoruyko S. End-to-end object detection with transformers. In: Proceedings of European Conference on Computer Vision (ECCV). Glasgow (2020). p. 213–29. doi: 10.1007/978-3-030-58452-8_13

[36] Zhao Y Lv W Xu S Wei J Wang G Dang Q . DETRs beat YOLOs on real-time object detection. In: Proceedings of the IEEE/CVF Conference on Computer Vision and Pattern Recognition (CVPR). Seattle, WA (2024). p. 16965–74. doi: 10.1109/CVPR52733.2024.01605

[37] Rasool N Iqbal Bhat J Ahmad Wani N Ahmad N Alshara MA. TransResUNet: revolutionizing glioma brain tumor segmentation through transformer-enhanced residual UNet. IEEE Access. (2024) 12:72105–16. doi: 10.1109/ACCESS.2024.3402947

[38] Dosovitskiy A Beyer L Kolesnikov A Weissenborn D Zhai X Unterthiner T . An image is worth 16 × 16 words: transformers for image recognition at scale. In: Proceedings of the International Conference on Learning Representations (ICLR). Virtual Event (2021).

[39] Meng D Chen X Fan Z Zeng G Li H Yuan Y . Conditional DETR for fast training convergence. In:Montreal QC, Editor. Proceedings of the IEEE/CVF International Conference on Computer Vision (ICCV). Montreal, QC (2021). p. 3651–60. doi: 10.1109/ICCV48922.2021.00363

[40] Tuteja P Wani MA Wani NA Bedi J. EASE-Net: an explainable AI-based segmentation and ensemble network for interpretable brain tumor diagnosis and classification in MRI images. Arab J Sci Eng. (2026). doi: 10.1007/s13369-026-11350-7

[41] Rasool N Wani NAA Bhat JI Saharan S Sharma VKK Alsulami BS . CNN-TumorNet: leveraging explainability in deep learning for precise brain tumor diagnosis on MRI images. Front. Oncol. (2025) 15:2025. doi: 10.3389/fonc.2025.1554559 PMC11979982 40206584

[42] Sajid Hussain S Wani NA Kaur J Ahmad N Ahmad S. Next-generation automation in neuro-oncology: advanced neural networks for MRI-based brain tumor segmentation and classification. IEEE Access. (2025) 13:41141–58. doi: 10.1109/ACCESS.2025.3547796

[43] Djoumessi K Mensah SO Berens P. A hybrid fully convolutional CNN-transformer model for inherently interpretable disease detection from retinal fundus images. In: Interpretability of Machine Intelligence in Medical Image Computing. Springer (2025). p. 106–16. doi: 10.1007/978-3-032-17611-0_11

